# Monitoring the Processing of Dry Fermented Sausages with a Portable NIRS Device

**DOI:** 10.3390/foods9091294

**Published:** 2020-09-14

**Authors:** Alberto González-Mohino, Trinidad Pérez-Palacios, Teresa Antequera, Jorge Ruiz-Carrascal, Lary Souza Olegario, Silvia Grassi

**Affiliations:** 1 Meat and Meat Products Research Institute (IProCar), Food Technology, University of Extremadura, 10003 Cáceres, Spain; albertogj@unex.es (A.G.-M.); triny@unex.es (T.P.-P.); tantero@unex.es (T.A.); jruiz@unex.es (J.R.-C.); 2 Department of Food Engineering, Technology Centre, Federal University of Paraiba, 58051-900 Joao Pessoa, Paraiba, Brazil; Laryolegario@hotmail.com; 3 Department of Food, Environmental, and Nutritional Sciences (DeFENS), Università degli Studi di Milano, via G. Celoria 2, 20133 Milan, Italy

**Keywords:** dry-fermented sausages, near infrared spectroscopy, portable device, PLS-DA

## Abstract

This work studies the ability of a MicroNIR (VIAVI, Santa Rosa, CA) device to monitor the dry fermented sausage process with the use of multivariate data analysis. Thirty sausages were made and subjected to dry fermentation, which was divided into four main stages. Physicochemical (weight lost, pH, moisture content, water activity, color, hardness, and thiobarbiruric reactive substances analysis) and sensory (quantitative descriptive analysis) characterizations of samples on different steps of the ripening process were performed. Near-infrared (NIR) spectra (950–1650 nm) were taken throughout the process at three points of the samples. Physicochemical data were explored by distance to K-Nearest Neighbor (K-NN) cluster analysis, while NIR spectra were studied by partial least square–discriminant analysis; before these models, Principal Component Analysis (PCA) was performed in both databases. The results of multivariate data analysis showed the ability to monitor and classify the different stages of ripening process (mainly the fermentation and drying steps). This study showed that a portable NIR device (MicroNIR) is a nondestructive, simple, noninvasive, fast, and cost-effective tool with the ability to monitor the dry fermented sausage processing and to classify samples as a function of the stage, constituting a feasible decision method for sausages to progress to the following processing stage.

## 1. Introduction

Dry-fermented meat products are one of the eldest and more remarkable groups of processed meats and constitute a key aspect in the identity, culture, and heritage of numerous regions. The great interest in traditional dry-fermented meats is specially remarkable in Europe, due not only to their great economic weight but also to their unique sensory features, which are a consequence of the raw material and the manufacturing process [1]. Traditional dry-fermented sausages are mostly manufactured with lean and fat from pork in small-scale production plants [2]. As other authors reported [3], most of these sausages are seasoned and processed with traditional manufacturing, the domestic environment being characterized by the limited degree of mechanization and final product control, which may bring higher heterogeneity to their quality. The implementation of control systems for the whole dry-fermented sausage process, ensuring the quality and safety of the product, would contribute to overcoming these issues. As most of traditional product industries are small-scale, such a control system should be simple, cheap, and easily implemented in fermenting-drying domestic or pilot-scale chambers.

Several analytical methods, such as physicochemical, chromatography, mass spectrometry, or sensory ones, have been shown to be able to collect accurate data from which the global quality of food products can be inferred. Nevertheless, these techniques are expensive, tedious, time- and solvent-consuming, and complex to use and require destruction of the sample.

Near-infrared (NIR) spectroscopy may well be an alternative to those other analysis methods, since it is cheaper than other instrumental techniques and allows for a nondestructive, simple, and fast analysis [4]. NIR spectroscopy combined with multivariate data analysis (chemometrics) have recently been demonstrated to be a solution of process analytical technology in the food industry [5]. Indeed, from NIR spectroscopy data, information about chemical, textural, and even microbiological compositions can be simultaneously inferred. Among the different chemometric methods that could be applied, Principal Component Analysis (PCA) and Partial Least Square-Discriminant Analysis (PLS-DA) became of interest, as they are able to take advantage of the structures in highly overlapping and colinear data [6].

Among the different commercially available NIR equipment, NIR handheld devices are a good alternative to benchtop instruments, being equally reliable but cheaper and faster and allowing in situ analyses. In fact, the advantages of NIR portable devices have been reported by other authors, highlighting cost reduction [7,8,9] as well as lower environmental impact [10] in comparison with benchtop ones. In addition, it is important to note that portable instruments are quite suitable for traditional product analysis, as reported in previous studies [11]. Portable NIR devices have been used to face different food-related issues, such as the determination of fish freshness [12] or the prediction of lycopene content in tomato [13]. Among different portable devices, MicroNIR is one of the most reliable due to its high resolution and broad spectral range. MicroNIR has been largely applied in several food matrices, including meat products [14], to assess dry cured ham quality parameters in dry cured ham [15], to monitor chicken meat authenticity [16], or to predict beef quality [17]. However, its use in monitoring the fermentation process in dry-fermented sausage has not been addressed yet.

Therefore, this work aims at developing a fast and noninvasive approach to monitor the process of traditional dry fermented sausages and to classify samples according to their processing stage by using a NIR spectroscopy handheld device coupled with multivariate data analysis.

## 2. Materials and Methods

### 2.1. Sausage Manufacturing

Twenty kilograms of pork lean and back fat (in proportion 4:1) were bought in a commercial supermarket (Mercadona, Caceres, Spain). Firstly, meat and fat were separately ground with a food grinding machine model PC-114 with a grinding plate of 4 mm (MAINCA, Equipamientos cárnicos S.L., Barcelona, España). Thereafter, the lean and fat were mixed by a mixing machine model RM-200 (MAINCA, Equipamientos cárnicos S.L., Barcelona, España) and added with the ingredients (salt (2.5% *w/w*), sucrose (0,75% *w/w*), garlic power (0,1%), spices (1% *w/w*), and sodium nitrite (100 ppm % *w/w*)). Subsequently, this mixture was stuffed into pork casings (35–40 mm diameter) of approximately 250 g each. Thus, 30 raw sausage samples were obtained. They were divided in two batches of 15 sausages (batch 1 and batch 2), which were subjected to the same fermentation and drying conditions but processed in different chambers.

The fermentation phase was carried out at 22–25 °C and 95% relative humidity (RH) for approximately 36 h until the pH reached 4.5. Thereafter, sausages were transferred into a drying-ripening chamber at 55 °C and 80% RH for 24 h (intense drying step) and continued drying at 15 °C for 60 h at an RH of 65%. The whole process took 120 h.

**Figure 1:**
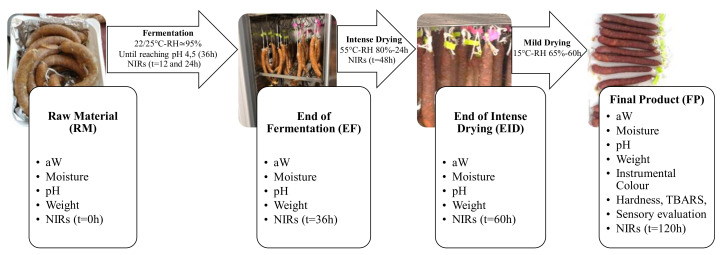
Scheme of the dry fermented sausage ripening process and analyses carried out at each stage. RH, relative humidity; aW, water activity; TBARS, thiobarbituric acid-reactive substances; NIRs, near infrared spectroscopy.

Figure 1 displays the processing conditions and the sampling. Sausages were analyzed by NIR spectroscopy at 0, 12, 24, 36, 48, 60, and 120 h. Moreover, three sausages for each processing step were used to perform destructive analyses to determine weight loss, water activity (aW), moisture content, and pH. The processing phases considered were the beginning of the processing (as raw material, RM), the end of fermentation (EF), and the intense drying (EID) stages. The rest of sausages (*n* = 6) finished the processing and were analyzed as a final product (FP). The determinations done in the FP batch were the same as the RW, EF, and EID ones but differed by adding instrumental color and texture, by lipid oxidation, and by sensory analysis.

### 2.2. Physicochemical Analysis (FQA)

Measurement of pH was determined in three different locations of each sausage (to obtain a representative averaged pH) with a meat pH meter electrode probe model FC232D (HANNA Instruments S.L., Eibar, Spain) equipped with automatic temperature compensation. The pH meter was calibrated with commercial buffer solutions (Crison, Barcelona, Spain) at pH 4.0 and 7.0 prior to use.

Moisture content was determined by drying the samples (5 g) at 102 °C following the procedure of the official methods of Association of Official Agricultural Chemists (AOAC International reference method 935.29) [18].

Water activity (aW) was determined by a water activity measuring equipment (Lab Master-aw; NOVASINA AG, Lachen, Switzerland).

Instrumental color of the sausages was measured using a portable reflectance spectrophotometer (Konica Minolta CM-600d, Osaka, Japan) that was calibrated with a standard white calibration tile. The analysis was carried out according to the principles laid down by the Commission International d’Eclairage (CIE) [19]. The following color coordinates were determined: lightness (L*), redness (a*), and yellowness (b*).

Instrumental texture (hardness) was analyzed by a texturometer TA XT-2i Texture Analyser (Stable Micro Systems Ltd., Surrey, UK). For each sample, five cubes (1 cm^3^) were obtained and analyzed. They were axially compressed to 50% of the original height with a flat plunger of 50 mm in diameter (P/50) at a crosshead speed of 2 mm × s^−1^ through a two-cycle sequence.

Lipid oxidation was measured by the thiobarbiruric reactive substances (TBARS) method, following the procedure described by Salih et al. [20] based on the concentration of malonaldehyde (MDA), and expressed as mg MDA/Kg sample.

These determinations were carried out in triplicate for each sample except for instrumental color and texture, which were analyzed in quintuplicate (due to the possible high variability of the samples and the laboratory error).

### 2.3. Sensory Analysis

Quantitative Descriptive Analysis (QDA) was carried out using 17 trained panelists (6 male and 11 female, age range 23–60 years). All of them were staff at the Meat and Meat Products Research Institute (IProCar) of the University of Extremadura (Spain). Attributes evaluated by the panel were selected based on attributes reported in previous studies with similar products and taking into consideration the consensus reached by a focus group of 6 panelists. The following attributes were chosen: red color intensity and cohesiveness for appearance; hardness, juiciness, and chewiness for texture; and flavor intensity, saltiness, spicy flavor persistence, and hot-spicy flavor. A 10-cm unstructured scale was used for attributes scoring, and verbal anchors were fixed as “extremely low” to “extremely high” for all evaluated attributes. Samples (one slice per plate) were served on glass plates with a glass of mineral water and a piece of unsalted cracker to follow the rinsing protocol between samples. Evaluations took place in individual booths under white fluorescence light. The serving order of the samples was randomized according to the Williams Latin Square design. FIZZ software 2.20 C version (Biosystemes, Couternon, France, 2002) was used for collecting the scores.

### 2.4. MicroNIR Analysis

**Figure 2:**
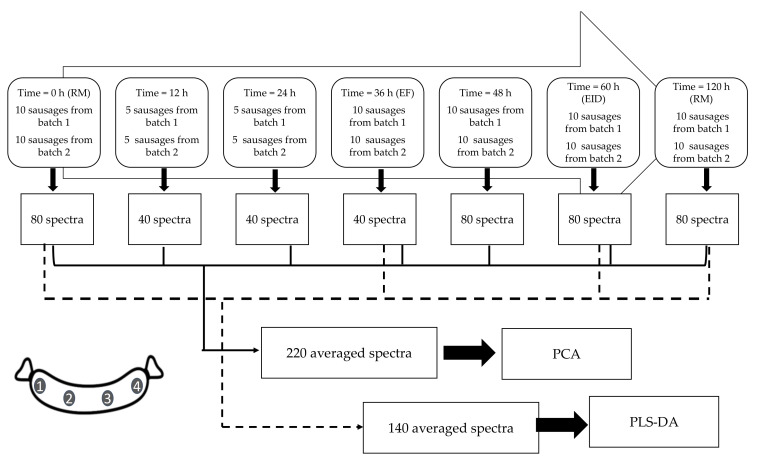
Scheme of the spectra acquisition procedure. RM, raw material; EF, the end of fermentation; end the intense drying (EID); FP, final product stages; PCA, Principal Component Analysis; and PLS-DA, Partial Least Square-Discriminant Analysis.

MicroNIR OnSite (VIAVI, Santa Rosa, CA) was used to analyze different sausages on the external surface of four different points equally spaced-out at each sampling time according to the scheme reported in Figure 2.

The MicroNIR spectral range was set to 950–1650 nm, with a 12.5 µs integration time and 200 scans, with a spectral bandwidth lower than 1.25% of center wavelength, typically 1% (e.g., at 1000 nm, the resolution is lower than 12.5 nm) and signal-to-noise ratio of 25,000.

Spectral acquisition was performed for 10 sausages selected from batch 1 and 10 from batch 2, every 12 h (i.e., at 0, 12, 24, 36, 48, and 60 h) and at the end of the process (120 h) for a total of seven sampling times and 440 spectra, as reported in Figure 2.

### 2.5. Data Analysis

Mean values and standard deviation were obtained from physicochemical and sensory data. Moreover, physicochemical results collected along the four stages of the process (weight losses, water activity, moisture, and pH) were explored by a Principal Component Analysis (PCA) to visualize the relationships among objects and variables with a biplot of scores and loadings. Thereafter, a clustering approach based on K-Nearest Neighbor’s algorithm (K-NN) was applied. K-NN is a simple nonlinear classification approach based on Euclidean distance and not requiring any assumptions on the underlying data distribution [21], and it is able to solve complex classification issues [22]. The K-NN algorithm was applied to discriminate clusters according to the sausages’ physicochemical characteristics to be later used as classes for the development of classification models based on NIR spectra.

Regarding MicroNIR, the four spectra collected for each sausage at each sampling time were averaged 2 by 2, thus merging the poles spectra and the ones collected closer to the longitudinal center. The averaged dataset (220 spectra × 125 wavelengths) was preprocessed to minimize the effect of noise and to enhance small but relevant spectral feature. Hence, the spectra dataset was transformed by smoothing (Savitzky-Golay, 3-wavelength gap size) followed by first derivative (Savitzky-Golay, 3-wavelength gap size and 2nd order polynomial) and mean center.

Data exploration by PCA lead to a reduction of the dataset due to outlier presence prior to developing a classification model by Partial Least Square Discriminant Analysis (PLS-DA). In order to do that, the 2-by-2 averaged spectra collected by MicroNIR for the sampling points corresponding to phases RM, EF, EID, and FP (140 spectra × 125 wavelengths) were split into a calibration set accounting for 66% of the data and a test set with the remaining 33% of samples. The PLS-DA method was selected to develop a classification model based on the NIR data according to the a priori classes defined by cluster analysis performed on the physicochemical data. A model was developed from the calibration dataset and internally validated by the Venetian blinds cross-validation procedure. Furthermore, the prediction capability was assessed by external validation using the test set, counting samples not used in the model development. According to Grassi et al. [21], PLS-DA was evaluated in all phases, i.e., calibration, cross-validation, and prediction, by two metrics, sensitivity (SENS) and specificity (SPEC), which are computed on the bases of four-factor (True Positive (TP), False Positive (FP), True Negative (TN), and False Negative (FN)). Thus, SENS describes the model capability to correctly recognize samples belonging to the considered class, whereas SPEC expresses the model capability to correctly reject samples belonging to all the other classes. Both metrics values are in a range from 0 (no correct prediction) to 1 (perfect classification).

Data analyses were performed under Matlab environment (R2017b, The Mathworks, Inc., Natick, MA, USA) eventually using the PLS toolbox v. 8.5 (Eigenvector Research, Inc., Manson, WA, USA) software package.

## 3. Results and Discussion

### 3.1. Physicochemical and Sensory Results

Table 1 shows, along the four stages, the mean averages and standards deviation of aW, moisture, pH, and weight loss of both dry-fermented sausages batches together, since no significant differences were found between them.

**Figure 3:**
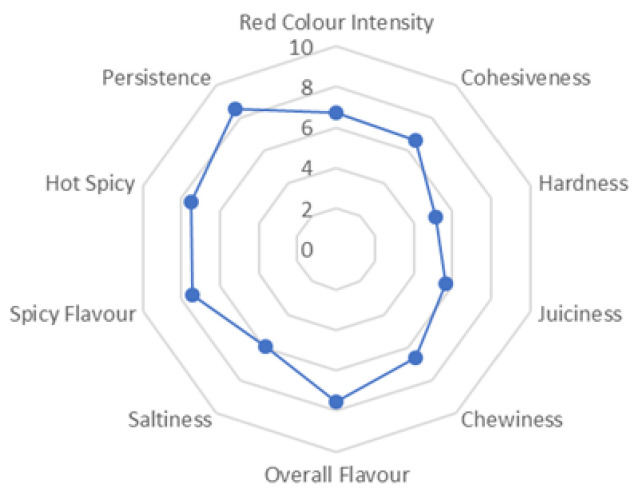
Results on sensory analysis from Quantitative Descriptive Analysis (QDA): the attributes are assessed within a 10-cm scale, from extremely low to extremely high.

As expected, weight loss increased through the process, which is crucial for this type of product [23]. As a consequence, moisture and aW also followed a decreasing trend in their values from the first phase (RM) to the successive stages of the dry fermented process, as has been previously described [24,25]. Concerning pH, it also underwent an initial decrease between the RM and EF phases but, thereafter, increased during the drying stages, which is a common pattern for this type of product [25]. In the final product, TBARS (0.23 ± 0.01), instrumental color L* (53.8 ± 1.4), a* (21.7 ± 2.2), b* (25.1 ± 4.1), instrumental hardness (133.5 ± 6.8 kg), and QDA attributes (represented in Figure 3) were also analyzed in order to check whether these dry fermented sausages fulfill the sensory and technological requirements for this type of product. Comparing the found values with those reported by other authors, parameters such as aW (ranged from 0.84 to 0.87) and pH (ranged from 5.59 to 5.89) were similar to reported values [26,27]. Moreover, color intensity and saltiness values were comparable with those reported in these two studies when considering control traditional samples. Nevertheless, pH values in our sausages did not experience an increase after the fermentation phase as high as in other sausages [27,28]. Moisture (33.8%) and weight loss (33.0%) values in samples from the last stage of ripening obtained by other authors were also comparable with our results, which validates the dehydration process, crucial for guaranteeing the preservation of these kinds of meat products [29]. Regarding aW, this parameter is crucial for the safety of this kind of product, since values below 0.9 ensure a stable product at room temperature, limiting the growth of spoilage and pathogenic bacteria [30]. The obtained values in the final product (0.875 ± 0.01) ensure the safety of our sausages.

If our traditional small-scale dry-fermented sausages are compared with commercial sausages, the results are coherent with those reported by Lopez et al. [1], who characterized ten commercial dry fermented sausages from a physicochemical and a sensory perspective, determining pH (ranged from 5.14 to 6.03), moisture (ranged from 28.75 to 48.70), and color parameters L* (ranged from 32.22 to 54.75), a* (ranged from 16.93 to 26.57), and b* (ranged from 6.42 to 15.91) of the commercial products.

**Figure 4:**
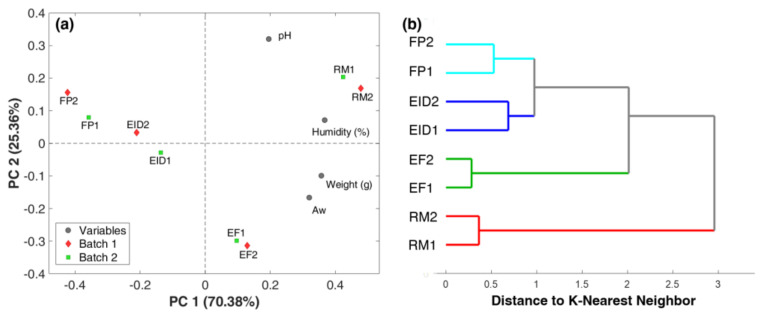
Physicochemical results: (**a**) biplot of Principal Component Analysis and (**b**) dendrogram of cluster analysis by K-nearest neighbor. Raw material, RM; end of fermentation, EF; end of intense drying, EID; and final product, FP.

Figure 4a displays the PCA biplot obtained for physicochemical data collected along the four steps of the dry-fermenting process for both batches, while Figure 4b displays the dendrogram obtained by cluster analysis through the K-nearest neighbor algorithm performed on the same dataset for both batches. The PCA biplot, defined by two first-principal components, accounts for 95.74% of the total variance (70.38 for PC1 and 25.36 for PC2). Both experimental batches were closely located for all stages of the processing. The RM group (t = 0 h, RM1 and RM2) is located in the I quadrant of the plot and correlates with pH and moisture content, both located in the same quadrant. Samples from the EF group (t = 36 h, EF1 and EF2) are situated in the IV quadrant, the same one where weight loss and aW are located. This location explains that PC2 differentiates samples according to changes mainly linked to pH, a parameter subjected to high variation during the fermentation process. Indeed, pH passes from an average of 5.7 to an average of 4.5 from RM to EF samples, respectively, thus distancing EF from the other samples and from the pH loadings. Samples from EID (t = 60, EID1 and EID2) and FP (t = 120, FP1 and FP2) are separated from RM and EF samples along PC1, showing highly negative PC1 scores. Their location is well explained by moisture content and weight variables, which, on the contrary, show high positive PC1 loadings. As a matter of fact, the drying process highly reduced moisture and weight of the sausages, especially in the intense drying step. Thus, EID and FP samples are characterized by similar PC1 scores but are quite different from RM and EF samples. The slight difference between EID and FP could be attributed to aW, for which the loadings call EID samples to slightly lower PC2 scores. PCA results on the physicochemical data could clearly separate three of the four stages of the dry fermented sausages process, though this analysis only constitutes a preliminary exploration. Further investigation by K-NN cluster clearly defined the existence of four groups according to the process phases at a K-NN distance of 1. In detail, Figure 4b reports the obtained dendrogram: RM1 and RM2 form a cluster differentiating from the other samples at 3 K-NN distance, the EF1 and EF2 cluster separates from the other samples at a distance of 2 K-NN, and EID1 and EID2 distinguish from FP1 and FP2 at a smaller distance (K-NN = 1), confirming the similarity observed by PCA.

### 3.2. Near Infrared Spectroscopy Results Exploration

**Figure 5:**
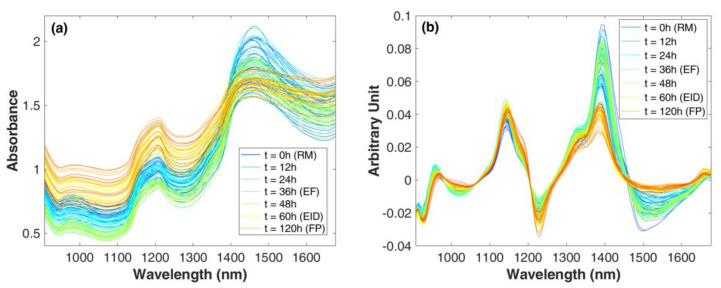
Near-infrared (NIR) spectra: (**a**) raw MicroNIR spectra and (**b**) MicroNIR spectra after first derivative transformation. Spectra are colored according to sampling time (h). Raw material, RM; end of fermentation, EF; end of intense drying, EID; and final product, FP.

NIR spectra acquired along the process are reported in Figure 5. The higher differences in the spectral absorptions are present around 930–1300 nm and 1150–1200 nm and from 1370 to 1650 nm. In these areas, absorption peaks of water are present: 979, 1200, and 1453 nm, corresponding to the first overtone of symmetric and asymmetric stretching, to a combination of stretching and bending, and to a combination of the stretching modes of OH bonds, respectively [31]. In particular, spectra acquired up to 48 h, i.e., before the drying phase, showed higher bands related to water absorption, whereas the loss of water during drying highly reduced the height of these bands. The reduction of absorption of the water bonds led to a better resolution of the shoulder present around 1150 nm, related to the second overtone of C–H [31] and possibly linked to the lipid fraction. The spectral changes are enhanced by transformation of the signal by smoothing and first derivative. In Figure 5b, it is possible to see how the spectra show a high variation along the considered range, discriminating the samples in two main groups, i.e., before and after the drying phases. Thus, it would be possible to establish the progress from one phase to the following visually according to spectra behavior. However, to better uncover the information hidden in the broad band characterizing the NIR spectra, it is necessary to use a multivariate approach.

**Figure 6:**
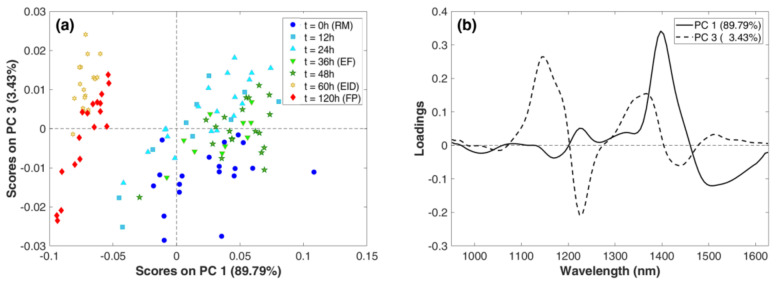
Principal Component Analysis on MicroNIR data: (**a**) score plots of PC1 vs. PC3 for samples colored according to sampling times and (**b**) loadings plot of PC1 and PC3.

Indeed, through PCA on the transformed spectral data, it was possible to unravel the relation between samples and variables. Figure 6a shows the PCA score plots defined by PC1 and PC3. First and third principal components accounted for 93.22% of the total variance (89.79 for PC1 and 3.43 for PC3). The sample distribution in the scores plot confirmed what was noticed in spectra visualization: the combination of PC1 and PC3 allowed for discrimination of samples before (from t = 0 to t = 48 h) and after the drying process (t ≥ 60 h).

As aforementioned, water content such as its activity is responsible for this effect, as observed in related loadings (Figure 6b). Indeed, the PC1 loadings highlighted the relevance of regions with maximums at 1224 and 1397 nm with positive effects and of regions with minimums at 1178 and 1497 nm with negative effects in the sample distribution. At the same extent, PC3 loadings are characterized by highly positive signal in the region with maximums at 1150 and 1360 nm and highly negative signal in the region with minimums at 1224 and 1435 nm. As other authors reported [32,33], NIR absorption is highly sensitive to variations in water content on meat, as occurred in our manufacturing process.

In the score plots (Figure 6a), a group of RM samples is distinguishable, which assumed negative PC3 scores, and it is mainly located in the IV quadrant. Samples collected between 12 and 48 h were more disperse, reproducing the inter-variability of the sausages along the process. Finally, spectra collected for samples undergoing the EID phase were well grouped in the II score quadrant (negative PC1 and positive P3), showing that the process absorbed the inter-variability of the sausages.

Furthermore, score distribution allowed the individuation of outliers, i.e., objects lying in an abnormal distance from observations of the same sampling time. This was the case for 4 samples for the RM and EID groups and 1 for the EF group.

### 3.3. PLS-DA Classification Models

The sample groups observed in the exploratory analysis did the groundwork for the development of classification models to discriminate samples by simple MicroNIR measures according to the process phase. For this purpose, the classes individuated by clustering analysis on the physicochemical results were used as a priori information (Y) to build PLS-DA classification models able to predict the ripening phase based on the MicroNIR data collected (X). The classification model was developed in calibration considering 88 of the collected spectra and validated both internally and externally by a prediction test set consisting of 44 spectra, i.e., by considering the 140 spectra collected for 20 sausages in phases RM, EF, EID, and FP net of the 8 outliers individuated by PCA. In Table 2, the metrics calculated for the model in cross-validation and prediction are reported. The PLS-DA model shows a successful classification ability as the RM, EID, and FP classes reached 1 for both sensitivity and specificity, proving the model capability to fully recognize samples belonging to the considered class (sensitivity = 1) together with the capability to correctly reject samples belonging to all other classes (specificity = 1). For example, reaching 1 for sensitivity means that all spectra belonging to the samples a priori identified as EID (ncal = 24 and npred = 12) were recognized by the PLS-DA model based on MicroNIR data as at the end of the intense drying process, as were the spectra belonging to RM, EF, and FP. The success of the classification model relies on the differences present in spectra. Indeed, the main differences observed in the spectral absorptions were present around 930–1300 nm and 1150–1200 nm and from 1370 to 1650 nm. In particular, changes were ascribed to peaks present at 979, 1200, and 1453 nm, which are linked to water absorption, the main compound changing during the process.

High specificity describes the perfect capability to correctly reject samples belonging to all other classes (specificity = 1). This was the case of the RM, EID, and FP classes, whereas for the EF cross-validation phase, it slowed to 0.98 as one spectrum, a priori defined as RM, was recognized by the classification model as EF. The misclassification could be linked to a higher moisture reduction on the pole points of one sausage at the RM phase, leading to a MicroNIR signal closer to the profile of an EF sample, i.e., with an higher absorption at peaks related to water presence (979, 1200, and 1453 nm). In any case, the prediction phase resulted in a perfect rejection of samples in all considered classes.

These results demonstrated that the data collected by the MicroNIR device allows for knowing the stage of the process, which may be useful to determine the passage from one stage to the next one.

To our knowledge, no other work developed classification models to determine the ripening stage during the dry fermented sausage process. Previously, NIR spectroscopy has been demonstrated to be a valid tool for the evaluation of the ripening phase of salami, a typical European dry fermented sausages [29]. However, in their study, the sampling procedure was destructive, 9 slices were analyzed for each investigated salami, and a benchtop instrument was employed, resulting in a higher spectral range (800–2800 nm). The authors succeeded in describing the ripening evolution by fitting the PC1 scores obtained from spectral data elaboration as a function of ripening time; however, no supervised modelling has been developed. Likewise, Gaitán-Jurado et al. [34] proposed a Visible-NIR (400–2000 nm)-based approach for online quality control of the packed slices of chorizo and salchichón (Spanish dry fermented sausages). Besides that, in this study, slices were considered, highlighting the effect of different factors, mainly plastic turns around the sample and slice thickness, affecting the sampling procedure and, thus, the prediction models. However, these authors developed only an approach intended for final product quality control, not considering food processing control, i.e., not evaluating the potential of NIR to model phenomena occurring during ripening.

Furthermore, other authors [35] developed partial least squares (PLS) regression models for water activity and moisture content prediction from slices of two types of fermented sausages. These authors demonstrated that, by prediction of the two parameters, it is possible to control the drying process of fermented sausages by NIR spectroscopymodels. However, the procedure developed considered only two out of multiple parameters influencing sausage processing, just the drying process, and it relied on a destructive procedure, as it required product slicing.

## 4. Conclusions

In this work, the feasibility of using a portable NIR device to monitor and classify the ripening process of dry fermented sausages using multivariate data analysis has been demonstrated.

Physicochemical (destructive) determinations allowed for the classification of dry fermented sausages according to their stage of processing by means of K-NN cluster analysis. Likewise, the nondestructive technique of NIR spectroscopy also enabled this classification, based on the visual observation of the spectra and by applying chemometrics on absorbance results, achieving high sensitivity and specificity classification values with PLS-DA.

Thus, the use of a nondestructive, portable, noninvasive, fast, easy-to-use and cost-effective tool, such as the MicroNIR device of this study, may be implemented to monitor the industrial processing of dry fermented sausages. This appears as a very interesting quality-control monitoring strategy specially for small and medium-sized companies, which usually lack equipped laboratories and analytical facilities. The implementation of such a procedure would allow for control of the progress from one stage to another or even to identify sausages that have reached the end of processing without the need for any other type of analysis. Further studies on different types of sausage processing may be necessary before upscaling this monitoring strategy to an industrial setup.

## Figures and Tables

**Table 1:** Moisture, aW, pH, and weight loss results along the four stages of the process as mean values and standard deviation of batches 1 and 2.

Stage	Moisture (%)	aW	pH	Weight Loss (%)
Raw Material (RM)	60.2 ± 0.8 ^1^	0.963 ± 0.01	5.72 ± 0.04	-
End of Fermentation (EF)	56.1 ± 1.7	0.961 ± 0.01	4.78 ± 0.11	10.2 ± 0.9
End of Intense Drying (EID)	41.9 ± 0.9	0.932 ± 0.02	5.17 ± 0.01	32.7 ± 1.7
Final Product (FP)	39.12 ± 0.8	0.875 ± 0.01	5.10 ± 0.07	35.8 ± 2.2

^1^ Mean values and standard deviation of batches 1 and 2.

**Table 2:** Results of PartialLleast Square–Discriminant analysis for sausage process stage identification: sensitivity (Sens) and specificity (Spec) values of models based on MicroNIR data. Raw material, RM; end of fermentation, EF; end of intense drying, EID; and final product, FP. In between brackets, the number of samples used for each single class for model calibration and prediction are given.

Validation Phase	*N* Sample	RM (24, 12)		EF (13, 6)		EID (24, 12)		FP (26, 14)	
		Sens	Spec	Sens	Spec	Sens	Spec	Sens	Spec
Cross-validation	88	1.00	1.00	1.00	0.98	1.00	1.00	1.00	1.00
Prediction	44	1.00	1.00	1.00	1.00	1.00	1.00	1.00	1.00

## References

[1] López C.M., Bru E., Vignolo G.M., Fadda S. (2012). Main Factors Affecting the Consumer Acceptance of Argentinean Fermented Sausages. J. Sens. Stud..

[2] Parunović N., Savic R., Radovic C. (2019). Qualitative properties of traditionally produced dry fermented sausages from meat of the autochthonous Mangalitsa pig breed. IOP Conf. Ser. Earth Environ. Sci..

[3] Talon R., Lebert I., Lebert A., Leroy S., Garriga M., Aymerich T., Drosinos E., Zanardi E., Ianieri A., Fraqueza M.J. (2007). Traditional dry fermented sausages produced in small-scale processing units in Mediterranean countries and Slovakia. 1: Microbial ecosystems of processing environments. Meat Sci..

[4] Giraudo A., Grassi S., Savorani F., Gavoci G., Casiraghi E., Geobaldo F. (2019). Determination of the geographical origin of green coffee beans using NIR spectroscopy and multivariate data analysis. Food Control..

[5] Grassi S., Alamprese C. (2018). Advances in NIR spectroscopy applied to process analytical technology in food industries. Curr. Opin. Food Sci..

[6] Feinberg M. (1990). Multivariate calibration, by H. Martens and T. Naes. TrAC Trends Anal. Chem..

[7] Barthès B., Kouakoua E., Clairotte M., Lallemand J., Chapuis-Lardy L., Rabenarivo M., Roussel S. (2019). Performance comparison between a miniaturized and a conventional near infrared reflectance (NIR) spectrometer for characterizing soil carbon and nitrogen. Geoderma.

[8] Yan H., Siesler H.W. (2018). Quantitative analysis of a pharmaceutical formulation: Performance comparison of different handheld near-infrared spectrometers. J. Pharm. Biomed. Anal..

[9] Serranti S., Bonifazi G., Gasbarrone R. (2018). Kiwifruits ripening assessment by portable hyperspectral devices. Sens. Agric. Food Quality Saf. X.

[10] Casson A., Beghi R., Giovenzana V., Fiorindo I., Tugnolo A., Guidetti R. (2020). Environmental advantages of visible and near infrared spectroscopy for the prediction of intact olive ripeness. Biosyst. Eng..

[11] Grassi S., Casiraghi E., Alamprese C. (2018). Handheld NIR device: A non-targeted approach to assess authenticity of fish fillets and patties. Food Chem..

[12] Moon E.J., Kim Y., Xu Y., Na Y., Giaccia A.J., Lee J.H. (2020). Evaluation of Salmon, Tuna, and Beef Freshness Using a Portable Spectrometer. Sensors.

[13] Goisser S., Wittmann S., Fernandes M., Mempel H., Ulrichs C. (2020). Comparison of colorimeter and different portable food-scanners for non-destructive prediction of lycopene content in tomato fruit. Postharvest Biol. Technol..

[14] Hassoun A., Måge I., Schmidt W.F., Temiz H.T., Li L., Kim H.-Y., Nilsen H., Biancolillo A., Aït-Kaddour A., Sikorski M. (2020). Fraud in Animal Origin Food Products: Advances in Emerging Spectroscopic Detection Methods over the Past Five Years. Foods.

[15] Pérez-Santaescolástica C., Fraeye I., Barba F.J., Gómez B., Tomasevic I., Romero A., Moreno A., Toldrá F., Lorenzo J.M. (2019). Application of non-invasive technologies in dry-cured ham: An overview. Trends Food Sci. Technol..

[16] Parastar H., Van Kollenburg G.H., Weesepoel Y., Doel A.V.D., Buydens L., Jansen J. (2020). Integration of handheld NIR and machine learning to “Measure & Monitor” chicken meat authenticity. Food Control..

[17] Savoia S., Albera A., Brugiapaglia A., Di Stasio L., Ferragina A., Cecchinato A., Bittante G. (2020). Prediction of meat quality traits in the abattoir using portable and hand-held near-infrared spectrometers. Meat Sci..

[18] Horwitz W. (2000). Official Methods of Analysis the of AOAC International.

[19] Billmeyer F.W., Saltzman M. (1981). Principles of Color Technology.

[20] Salih A.M., Smith D.M., Price J.F., Dawson L.E. (1987). Modified Extraction 2-Thiobarbituric Acid Method for Measuring Lipid Oxidation in Poultry. Poult. Sci..

[21] Grassi S., Benedetti S., Opizzio M., Di Nardo E., Buratti S. (2019). Nardo Meat and Fish Freshness Assessment by a Portable and Simplified Electronic Nose System (Mastersense). Sensors.

[22] Oliveri P., Malegori C., Casale M., Tartacca E., Salvatori G. (2019). An innovative multivariate strategy for HSI-NIR images to automatically detect defects in green coffee. Talanta.

[23] Cavalheiro C.P., Ruiz-Capillas C., Herrero A.M., Jiménez-Colmenero F., Pintado T., Fries L.L.M., De Menezes C.R. (2019). Effect of different strategies of Lactobacillus plantarum incorporation in chorizo sausages. J. Sci. Food Agric..

[24] Hu Y., Zhang L., Zhang H., Wang Y., Chen Q., Kong B. (2020). Physicochemical properties and flavour profile of fermented dry sausages with a reduction of sodium chloride. LWT.

[25] Balamurugan S., Gemmell C., Lau A.T.Y., Arvaj L., Strange P., Gao A., Barbut S. (2020). High pressure processing during drying of fermented sausages can enhance safety and reduce time required to produce a dry fermented product. Food Control..

[26] Mendoza E., García M., Casas C., Selgas M. (2001). Inulin as fat substitute in low fat, dry fermented sausages. Meat Sci..

[27] Talon R., Leroy S., Lebert I., Giammarinaro P., Chacornac J., Latorre-Moratalla M., Vidalcarou C., Zanardi E., Conter M., Lebecque A. (2008). Safety improvement and preservation of typical sensory qualities of traditional dry fermented sausages using autochthonous starter cultures. Int. J. Food Microbiol..

[28] Aymerich T., Martin B., Garriga M., Vidal-Carou M., Bover-Cid S., Hugas M. (2006). Safety properties and molecular strain typing of lactic acid bacteria from slightly fermented sausages. J. Appl. Microbiol..

[29] Alamprese C., Fongaro L., Casiraghi E. (2016). Effect of fresh pork meat conditioning on quality characteristics of salami. Meat Sci..

[30] Taormina P.J., Sofos J.N., Gurtler J.B., Doyle M.B., Kornacki J.L. (2014). Low-Water Activity Meat Products. The Microbiological Safety of Low Water Activity Foods and Spices.

[31] Workman J., Weyer L. (2007). Practical Guide to Interpretive Near-Infrared Spectroscopy.

[32] Büning-Pfaue H. (2003). Analysis of water in food by near infrared spectroscopy. Food Chem..

[33] Ishikawa D., Ueno G., Fujii T. (2017). Estimation Method of Moisture Content at the Meat Surface During Drying Process by NIR Spectroscopy and Its Application for Monitoring of Water Activity. Jpn. J. Food Eng..

[34] Gaitán-Jurado A.J., Rincón F., Ortiz-Somovilla V., España-España F. (2008). Detection of Factors Affecting the Acquisition of Visible-Near Infrared Fibre-Optic Probe Spectra of Commercial Meat Products. J. Near Infrared Spectrosc..

[35] Collell C., Gou P., Arnau J., Muñoz I., Comaposada J. (2012). NIR technology for on-line determination of superficial aw and moisture content during the drying process of fermented sausages. Food Chem..

